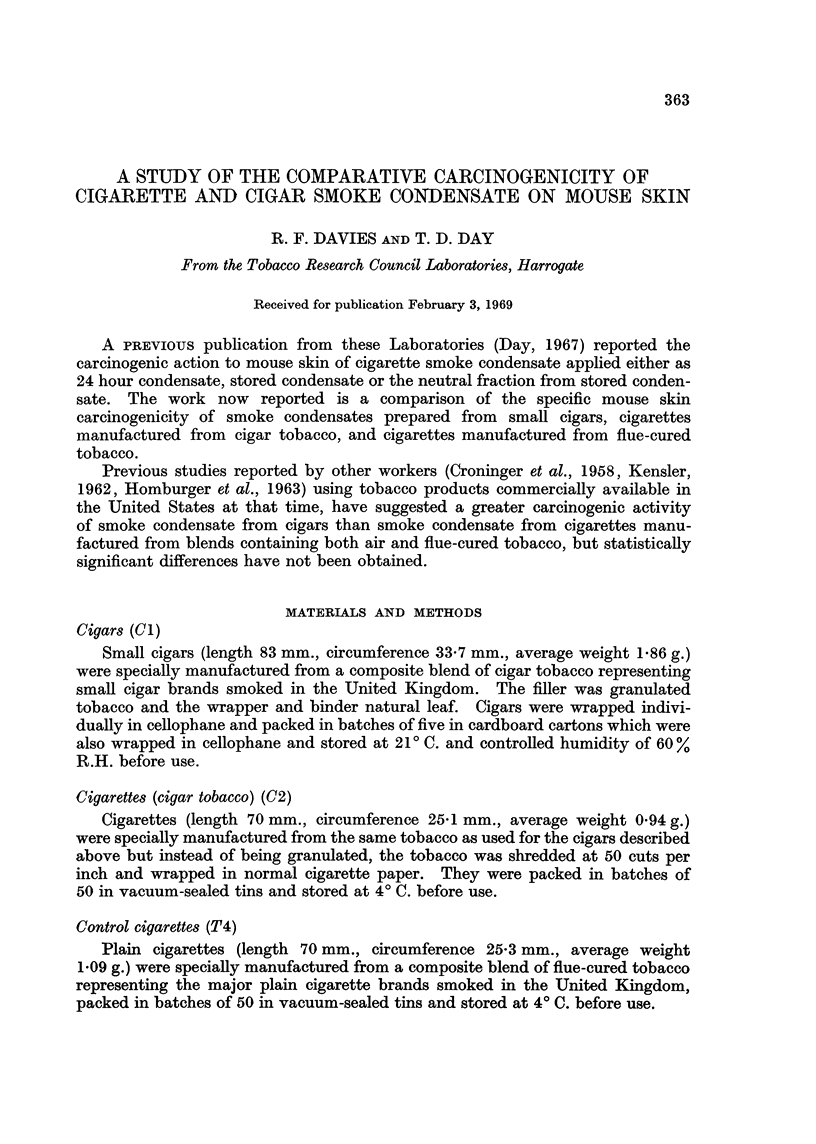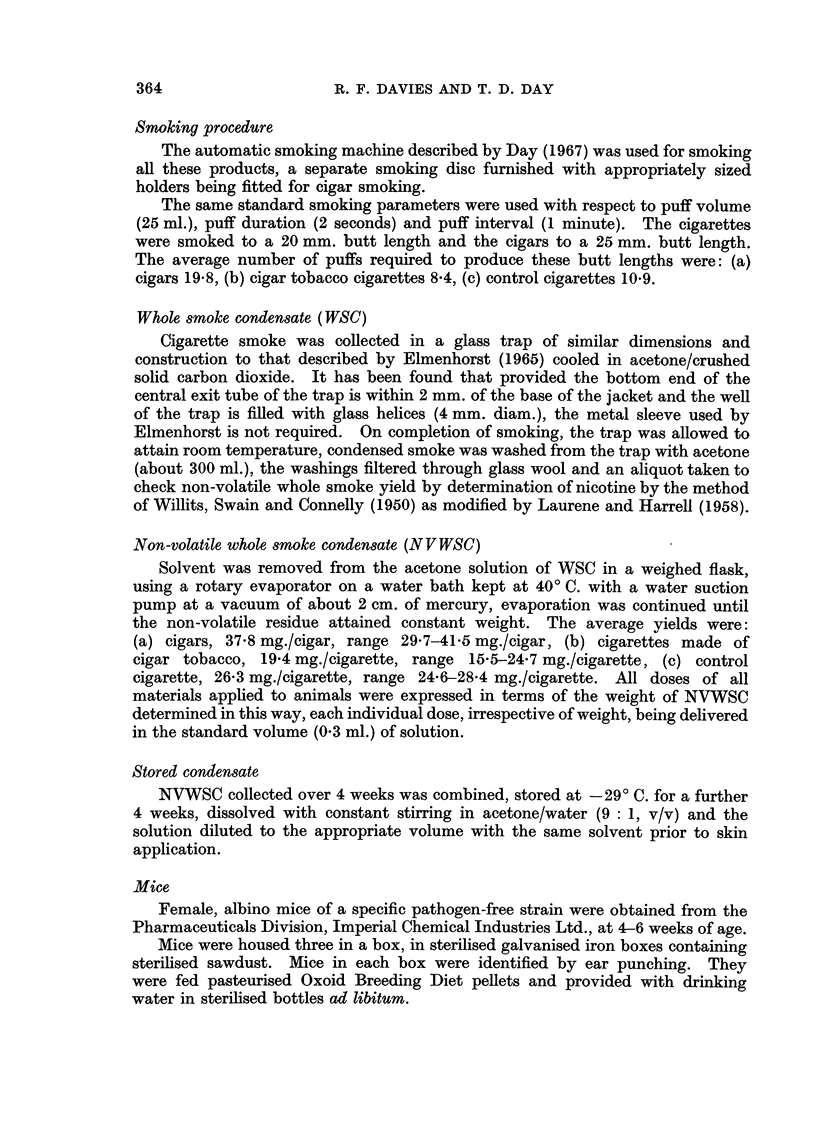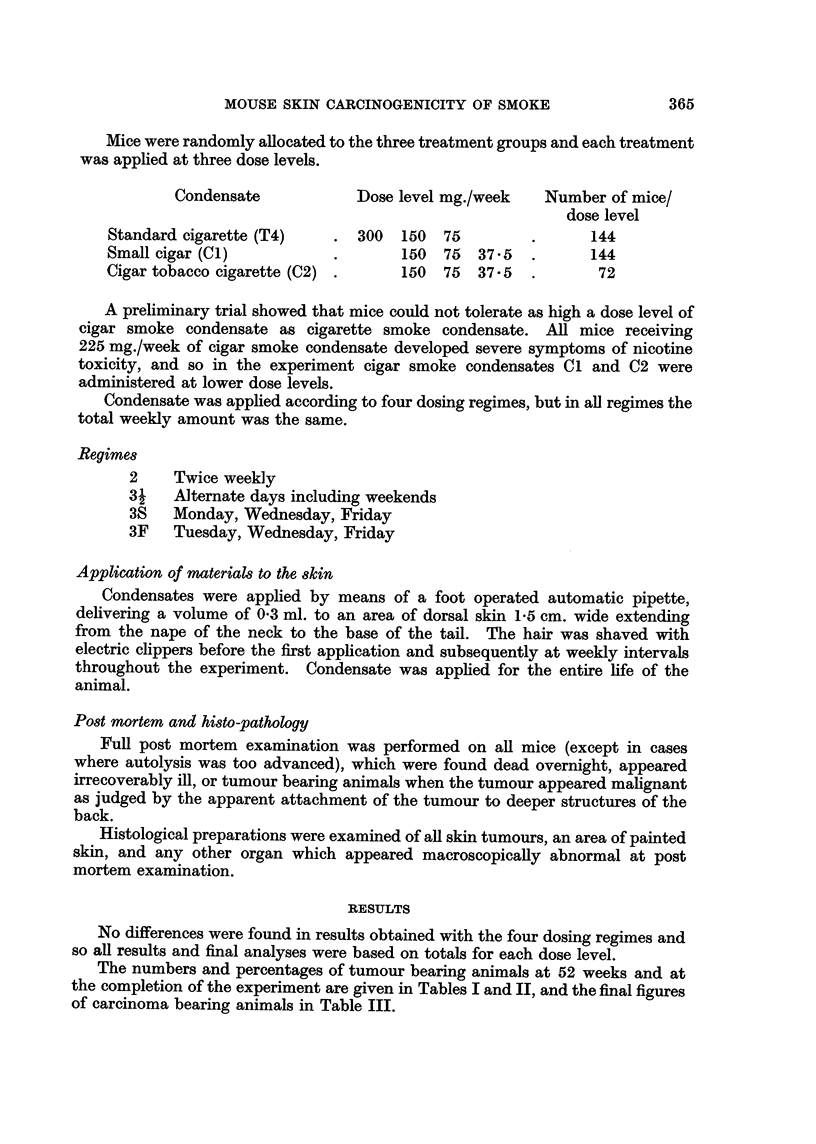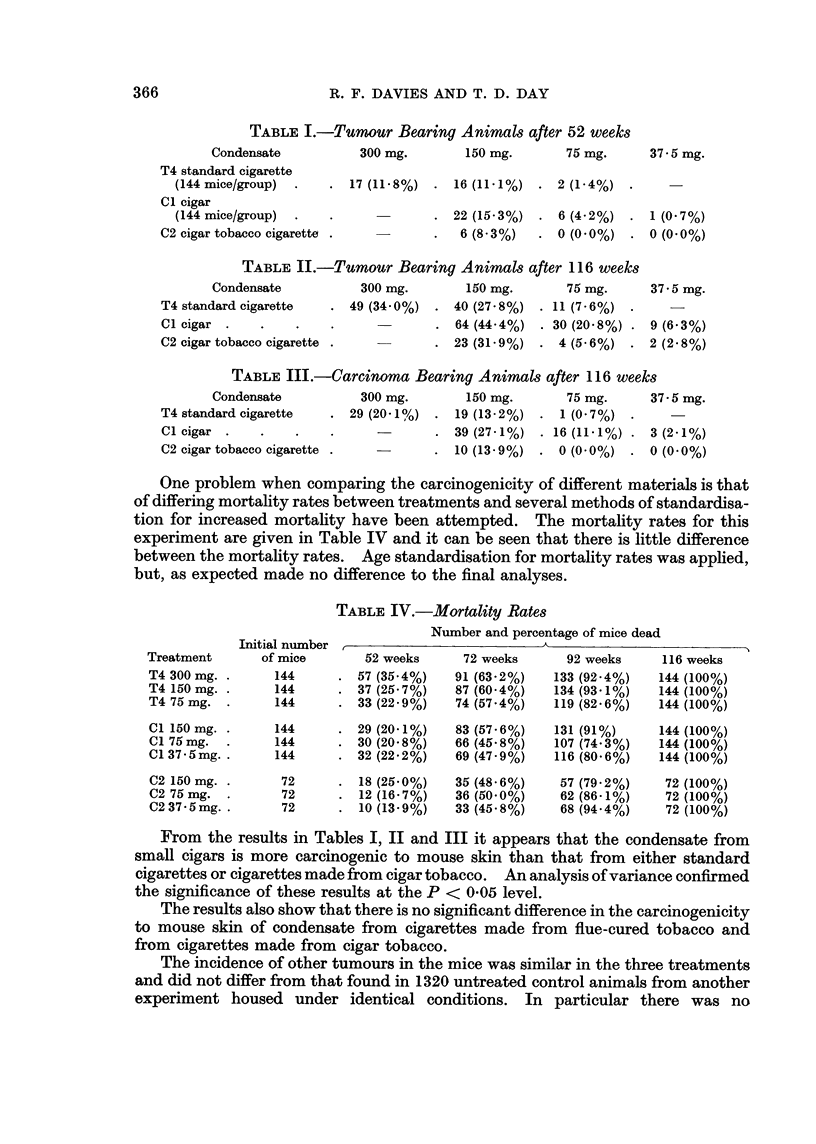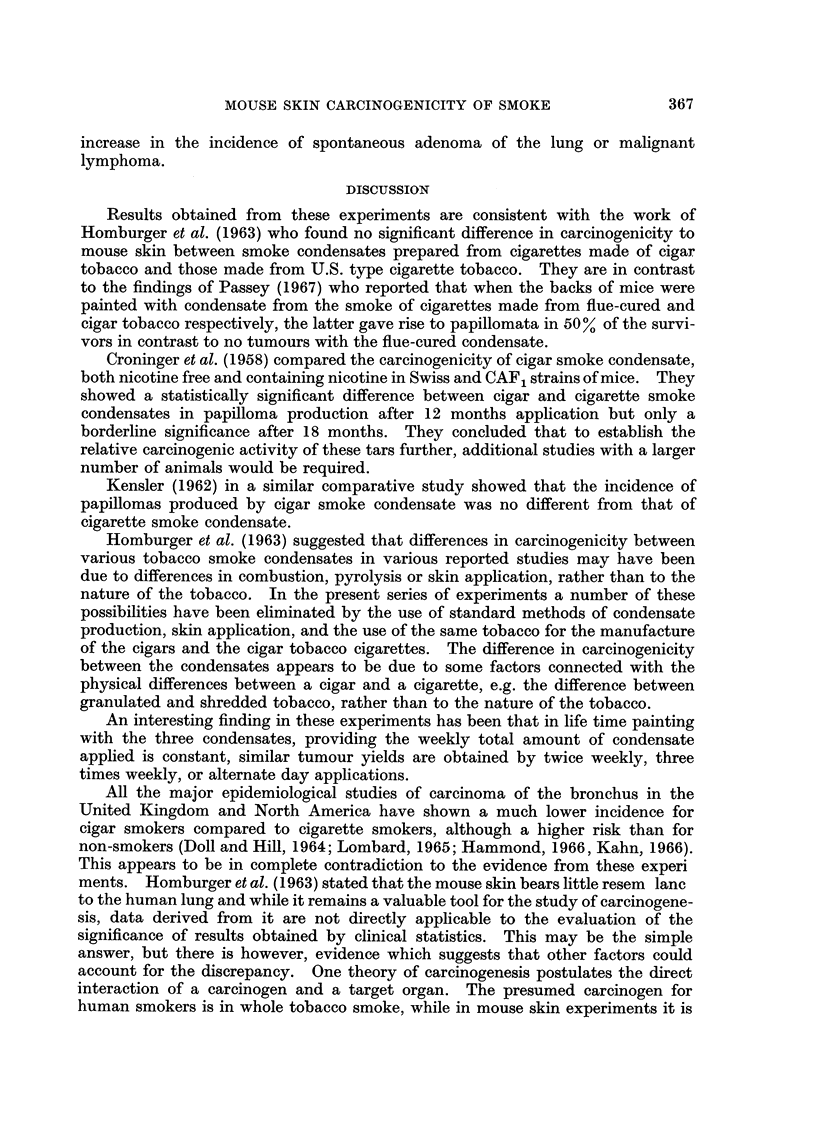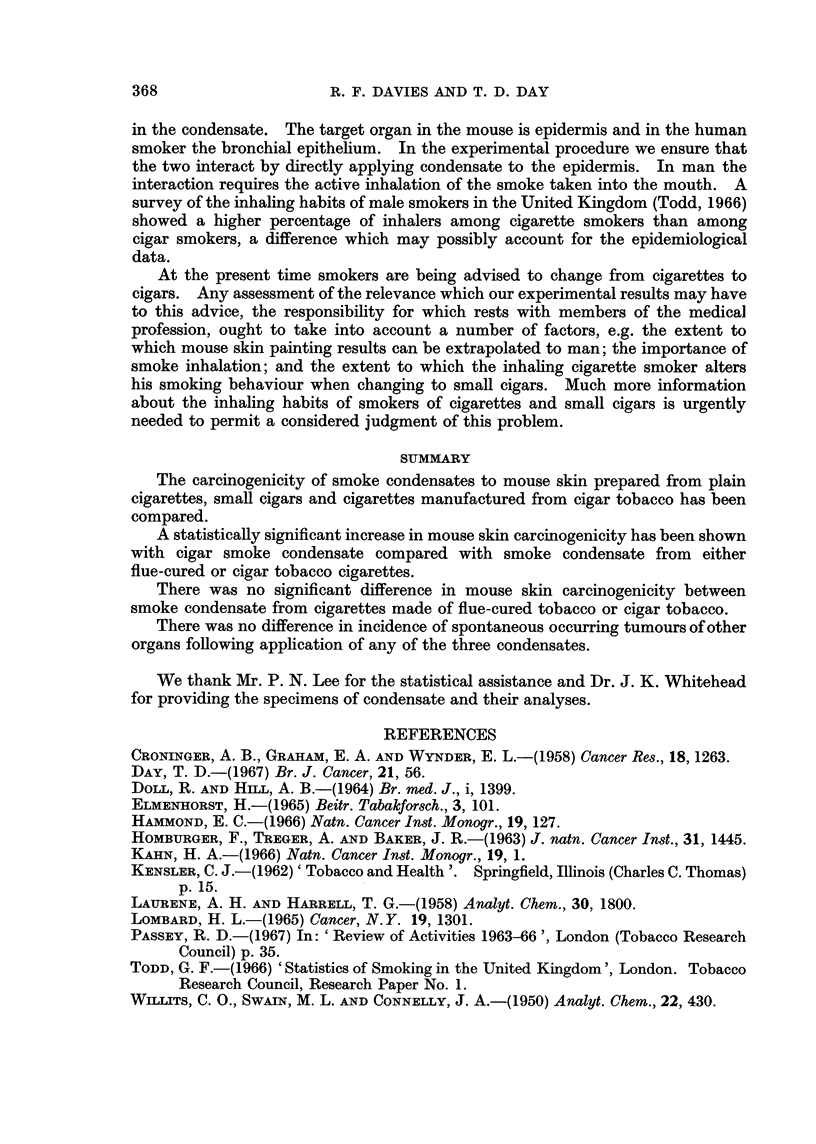# A study of the comparative carcinogenicity of cigarette and cigar smoke condensate on mouse skin.

**DOI:** 10.1038/bjc.1969.47

**Published:** 1969-06

**Authors:** R. F. Davies, T. D. Day


### A STUDY OF THE COMPARATIVE CARCINOGENICITY OF CIGARETTE AND CIGAR SMOKE CONDENSATE ON MOUSE SKIN

R. F. DAVIES AND T. D. DAY

*From the Tobacco Research Council Laboratories, Harrogate*

Received for publication February 3, 1969

A PREVIOUS publication from these Laboratories (Day, 1967) reported the carcinogenic action to mouse skin of cigarette smoke condensate applied either as 24 hour condensate, stored condensate or the neutral fraction from stored condensate. The work now reported is a comparison of the specific mouse skin carcinogenicity of smoke condensates prepared from small cigars, cigarettes manufactured from cigar tobacco, and cigarettes manufactured from flue-cured tobacco.

Previous studies reported by other workers (Croninger *et al.*, 1958, Kensler, 1962, Homburger *et al.*, 1963) using tobacco products commercially available in the United States at that time, have suggested a greater carcinogenic activity of smoke condensate from cigars than smoke condensate from cigarettes manufactured from blends containing both air and flue-cured tobacco, but statistically significant differences have not been obtained.

### MATERIALS AND METHODS

### *Cigars* (*C*1)

Small cigars (length 83 mm., circumference 33·7 mm., average weight 1·86 g.) were specially manufactured from a composite blend of cigar tobacco representing small cigar brands smoked in the United Kingdom. The filler was granulated tobacco and the wrapper and binder natural leaf. Cigars were wrapped individually in cellophane and packed in batches of five in cardboard cartons which were also wrapped in cellophane and stored at 21° C. and controlled humidity of 60% R.H. before use.

### *Cigarettes* (*cigar tobacco*) (*C*2)

Cigarettes (length 70 mm., circumference 25·1 mm., average weight 0·94 g.) were specially manufactured from the same tobacco as used for the cigars described above but instead of being granulated, the tobacco was shredded at 50 cuts per inch and wrapped in normal cigarette paper. They were packed in batches of 50 in vacuum-sealed tins and stored at 4° C. before use.

### *Control cigarettes* (*T*4)

Plain cigarettes (length 70 mm., circumference 25·3 mm., average weight 1·09 g.) were specially manufactured from a composite blend of flue-cured tobacco representing the major plain cigarette brands smoked in the United Kingdom, packed in batches of 50 in vacuum-sealed tins and stored at 4° C. before use.

*Smoking procedure*

The automatic smoking machine described by Day (1967) was used for smoking all these products, a separate smoking disc furnished with appropriately sized holders being fitted for cigar smoking.

The same standard smoking parameters were used with respect to puff volume (25 ml.), puff duration (2 seconds) and puff interval (1 minute). The cigarettes were smoked to a 20 mm. butt length and the cigars to a 25 mm. butt length. The average number of puffs required to produce these butt lengths were: (a) cigars 19·8, (b) cigar tobacco cigarettes 8·4, (c) control cigarettes 10·9.

*Whole smoke condensate (WSC)*

Cigarette smoke was collected in a glass trap of similar dimensions and construction to that described by Elmenhorst (1965) cooled in acetone/crushed solid carbon dioxide. It has been found that provided the bottom end of the central exit tube of the trap is within 2 mm. of the base of the jacket and the well of the trap is filled with glass helices (4 mm. diam.), the metal sleeve used by Elmenhorst is not required. On completion of smoking, the trap was allowed to attain room temperature, condensed smoke was washed from the trap with acetone (about 300 ml.), the washings filtered through glass wool and an aliquot taken to check non-volatile whole smoke yield by determination of nicotine by the method of Willits, Swain and Connelly (1950) as modified by Laurene and Harrell (1958).

*Non-volatile whole smoke condensate (NVWSC)*

Solvent was removed from the acetone solution of WSC in a weighed flask, using a rotary evaporator on a water bath kept at 40° C. with a water suction pump at a vacuum of about 2 cm. of mercury, evaporation was continued until the non-volatile residue attained constant weight. The average yields were: (a) cigars, 37·8 mg./cigar, range 29·7–41·5 mg./cigar, (b) cigarettes made of cigar tobacco, 19·4 mg./cigarette, range 15·5–24·7 mg./cigarette, (c) control cigarette, 26·3 mg./cigarette, range 24·6–28·4 mg./cigarette. All doses of all materials applied to animals were expressed in terms of the weight of NVWSC determined in this way, each individual dose, irrespective of weight, being delivered in the standard volume (0·3 ml.) of solution.

*Stored condensate*

NVWSC collected over 4 weeks was combined, stored at −29° C. for a further 4 weeks, dissolved with constant stirring in acetone/water (9 : 1, v/v) and the solution diluted to the appropriate volume with the same solvent prior to skin application.

*Mice*

Female, albino mice of a specific pathogen-free strain were obtained from the Pharmaceuticals Division, Imperial Chemical Industries Ltd., at 4–6 weeks of age.

Mice were housed three in a box, in sterilised galvanised iron boxes containing sterilised sawdust. Mice in each box were identified by ear punching. They were fed pasteurised Oxoid Breeding Diet pellets and provided with drinking water in sterilised bottles *ad libitum*.

Mice were randomly allocated to the three treatment groups and each treatment was applied at three dose levels.

| Condensate | Dose level mg./week | | | | Number of mice/ dose level |
|---|---|---|---|---|---|
| Standard cigarette (T4) | 300 | 150 | 75 | | 144 |
| Small cigar (C1) | | 150 | 75 | 37·5 | 144 |
| Cigar tobacco cigarette (C2) | | 150 | 75 | 37·5 | 72 |

A preliminary trial showed that mice could not tolerate as high a dose level of cigar smoke condensate as cigarette smoke condensate. All mice receiving 225 mg./week of cigar smoke condensate developed severe symptoms of nicotine toxicity, and so in the experiment cigar smoke condensates C1 and C2 were administered at lower dose levels.

Condensate was applied according to four dosing regimes, but in all regimes the total weekly amount was the same.

*Regimes*

| | |
|---|---|
| 2 | Twice weekly |
| 3½ | Alternate days including weekends |
| 3S | Monday, Wednesday, Friday |
| 3F | Tuesday, Wednesday, Friday |

*Application of materials to the skin*

Condensates were applied by means of a foot operated automatic pipette, delivering a volume of 0·3 ml. to an area of dorsal skin 1·5 cm. wide extending from the nape of the neck to the base of the tail. The hair was shaved with electric clippers before the first application and subsequently at weekly intervals throughout the experiment. Condensate was applied for the entire life of the animal.

*Post mortem and histo-pathology*

Full post mortem examination was performed on all mice (except in cases where autolysis was too advanced), which were found dead overnight, appeared irrecoverably ill, or tumour bearing animals when the tumour appeared malignant as judged by the apparent attachment of the tumour to deeper structures of the back.

Histological preparations were examined of all skin tumours, an area of painted skin, and any other organ which appeared macroscopically abnormal at post mortem examination.

### RESULTS

No differences were found in results obtained with the four dosing regimes and so all results and final analyses were based on totals for each dose level.

The numbers and percentages of tumour bearing animals at 52 weeks and at the completion of the experiment are given in Tables I and II, and the final figures of carcinoma bearing animals in Table III.

TABLE I.—*Tumour Bearing Animals after* 52 *weeks*

| Condensate | 300 mg. | 150 mg. | 75 mg. | 37·5 mg. |
|---|---|---|---|---|
| T4 standard cigarette (144 mice/group) | 17 (11·8%) | 16 (11·1%) | 2 (1·4%) | — |
| C1 cigar (144 mice/group) | — | 22 (15·3%) | 6 (4·2%) | 1 (0·7%) |
| C2 cigar tobacco cigarette | — | 6 (8·3%) | 0 (0·0%) | 0 (0·0%) |

TABLE II.—*Tumour Bearing Animals after* 116 *weeks*

| Condensate | 300 mg. | 150 mg. | 75 mg. | 37·5 mg. |
|---|---|---|---|---|
| T4 standard cigarette | 49 (34·0%) | 40 (27·8%) | 11 (7·6%) | — |
| C1 cigar | — | 64 (44·4%) | 30 (20·8%) | 9 (6·3%) |
| C2 cigar tobacco cigarette | — | 23 (31·9%) | 4 (5·6%) | 2 (2·8%) |

TABLE III.—*Carcinoma Bearing Animals after* 116 *weeks*

| Condensate | 300 mg. | 150 mg. | 75 mg. | 37·5 mg. |
|---|---|---|---|---|
| T4 standard cigarette | 29 (20·1%) | 19 (13·2%) | 1 (0·7%) | — |
| C1 cigar | — | 39 (27·1%) | 16 (11·1%) | 3 (2·1%) |
| C2 cigar tobacco cigarette | — | 10 (13·9%) | 0 (0·0%) | 0 (0·0%) |

One problem when comparing the carcinogenicity of different materials is that of differing mortality rates between treatments and several methods of standardisation for increased mortality have been attempted. The mortality rates for this experiment are given in Table IV and it can be seen that there is little difference between the mortality rates. Age standardisation for mortality rates was applied, but, as expected made no difference to the final analyses.

TABLE IV.—*Mortality Rates*

| Treatment | Initial number of mice | Number and percentage of mice dead | | | |
|---|---|---|---|---|---|
| | | 52 weeks | 72 weeks | 92 weeks | 116 weeks |
| T4 300 mg. | 144 | 57 (35·4%) | 91 (63·2%) | 133 (92·4%) | 144 (100%) |
| T4 150 mg. | 144 | 37 (25·7%) | 87 (60·4%) | 134 (93·1%) | 144 (100%) |
| T4 75 mg. | 144 | 33 (22·9%) | 74 (57·4%) | 119 (82·6%) | 144 (100%) |
| C1 150 mg. | 144 | 29 (20·1%) | 83 (57·6%) | 131 (91%) | 144 (100%) |
| C1 75 mg. | 144 | 30 (20·8%) | 66 (45·8%) | 107 (74·3%) | 144 (100%) |
| C1 37·5 mg. | 144 | 32 (22·2%) | 69 (47·9%) | 116 (80·6%) | 144 (100%) |
| C2 150 mg. | 72 | 18 (25·0%) | 35 (48·6%) | 57 (79·2%) | 72 (100%) |
| C2 75 mg. | 72 | 12 (16·7%) | 36 (50·0%) | 62 (86·1%) | 72 (100%) |
| C2 37·5 mg. | 72 | 10 (13·9%) | 33 (45·8%) | 68 (94·4%) | 72 (100%) |

From the results in Tables I, II and III it appears that the condensate from small cigars is more carcinogenic to mouse skin than that from either standard cigarettes or cigarettes made from cigar tobacco. An analysis of variance confirmed the significance of these results at the $P < 0{\cdot}05$ level.

The results also show that there is no significant difference in the carcinogenicity to mouse skin of condensate from cigarettes made from flue-cured tobacco and from cigarettes made from cigar tobacco.

The incidence of other tumours in the mice was similar in the three treatments and did not differ from that found in 1320 untreated control animals from another experiment housed under identical conditions. In particular there was no increase in the incidence of spontaneous adenoma of the lung or malignant lymphoma.

### DISCUSSION

Results obtained from these experiments are consistent with the work of Homburger *et al.* (1963) who found no significant difference in carcinogenicity to mouse skin between smoke condensates prepared from cigarettes made of cigar tobacco and those made from U.S. type cigarette tobacco. They are in contrast to the findings of Passey (1967) who reported that when the backs of mice were painted with condensate from the smoke of cigarettes made from flue-cured and cigar tobacco respectively, the latter gave rise to papillomata in 50% of the survivors in contrast to no tumours with the flue-cured condensate.

Croninger *et al.* (1958) compared the carcinogenicity of cigar smoke condensate, both nicotine free and containing nicotine in Swiss and $CAF_1$ strains of mice. They showed a statistically significant difference between cigar and cigarette smoke condensates in papilloma production after 12 months application but only a borderline significance after 18 months. They concluded that to establish the relative carcinogenic activity of these tars further, additional studies with a larger number of animals would be required.

Kensler (1962) in a similar comparative study showed that the incidence of papillomas produced by cigar smoke condensate was no different from that of cigarette smoke condensate.

Homburger *et al.* (1963) suggested that differences in carcinogenicity between various tobacco smoke condensates in various reported studies may have been due to differences in combustion, pyrolysis or skin application, rather than to the nature of the tobacco. In the present series of experiments a number of these possibilities have been eliminated by the use of standard methods of condensate production, skin application, and the use of the same tobacco for the manufacture of the cigars and the cigar tobacco cigarettes. The difference in carcinogenicity between the condensates appears to be due to some factors connected with the physical differences between a cigar and a cigarette, e.g. the difference between granulated and shredded tobacco, rather than to the nature of the tobacco.

An interesting finding in these experiments has been that in life time painting with the three condensates, providing the weekly total amount of condensate applied is constant, similar tumour yields are obtained by twice weekly, three times weekly, or alternate day applications.

All the major epidemiological studies of carcinoma of the bronchus in the United Kingdom and North America have shown a much lower incidence for cigar smokers compared to cigarette smokers, although a higher risk than for non-smokers (Doll and Hill, 1964; Lombard, 1965; Hammond, 1966, Kahn, 1966). This appears to be in complete contradiction to the evidence from these experi ments. Homburger *et al.* (1963) stated that the mouse skin bears little resem lanc to the human lung and while it remains a valuable tool for the study of carcinogenesis, data derived from it are not directly applicable to the evaluation of the significance of results obtained by clinical statistics. This may be the simple answer, but there is however, evidence which suggests that other factors could account for the discrepancy. One theory of carcinogenesis postulates the direct interaction of a carcinogen and a target organ. The presumed carcinogen for human smokers is in whole tobacco smoke, while in mouse skin experiments it is in the condensate. The target organ in the mouse is epidermis and in the human smoker the bronchial epithelium. In the experimental procedure we ensure that the two interact by directly applying condensate to the epidermis. In man the interaction requires the active inhalation of the smoke taken into the mouth. A survey of the inhaling habits of male smokers in the United Kingdom (Todd, 1966) showed a higher percentage of inhalers among cigarette smokers than among cigar smokers, a difference which may possibly account for the epidemiological data.

At the present time smokers are being advised to change from cigarettes to cigars. Any assessment of the relevance which our experimental results may have to this advice, the responsibility for which rests with members of the medical profession, ought to take into account a number of factors, e.g. the extent to which mouse skin painting results can be extrapolated to man; the importance of smoke inhalation; and the extent to which the inhaling cigarette smoker alters his smoking behaviour when changing to small cigars. Much more information about the inhaling habits of smokers of cigarettes and small cigars is urgently needed to permit a considered judgment of this problem.

SUMMARY

The carcinogenicity of smoke condensates to mouse skin prepared from plain cigarettes, small cigars and cigarettes manufactured from cigar tobacco has been compared.

A statistically significant increase in mouse skin carcinogenicity has been shown with cigar smoke condensate compared with smoke condensate from either flue-cured or cigar tobacco cigarettes.

There was no significant difference in mouse skin carcinogenicity between smoke condensate from cigarettes made of flue-cured tobacco or cigar tobacco.

There was no difference in incidence of spontaneous occurring tumours of other organs following application of any of the three condensates.

We thank Mr. P. N. Lee for the statistical assistance and Dr. J. K. Whitehead for providing the specimens of condensate and their analyses.

### REFERENCES

Croninger, A. B., Graham, E. A. and Wynder, E. L.—(1958) *Cancer Res.*, **18**, 1263.
Day, T. D.—(1967) *Br. J. Cancer*, **21**, 56.
Doll, R. and Hill, A. B.—(1964) *Br. med. J.*, i, 1399.
Elmenhorst, H.—(1965) *Beitr. Tabakforsch.*, **3**, 101.
Hammond, E. C.—(1966) *Natn. Cancer Inst. Monogr.*, **19**, 127.
Homburger, F., Treger, A. and Baker, J. R.—(1963) *J. natn. Cancer Inst.*, **31**, 1445.
Kahn, H. A.—(1966) *Natn. Cancer Inst. Monogr.*, **19**, 1.
Kensler, C. J.—(1962) 'Tobacco and Health'. Springfield, Illinois (Charles C. Thomas) p. 15.
Laurene, A. H. and Harrell, T. G.—(1958) *Analyt. Chem.*, **30**, 1800.
Lombard, H. L.—(1965) *Cancer, N.Y.* **19**, 1301.
Passey, R. D.—(1967) In: 'Review of Activities 1963–66', London (Tobacco Research Council) p. 35.
Todd, G. F.—(1966) 'Statistics of Smoking in the United Kingdom', London. Tobacco Research Council, Research Paper No. 1.
Willits, C. O., Swain, M. L. and Connelly, J. A.—(1950) *Analyt. Chem.*, **22**, 430.